# Outer membrane vesicles from bacteria: Role and potential value in the pathogenesis of chronic respiratory diseases

**DOI:** 10.3389/fcimb.2022.1093327

**Published:** 2022-12-08

**Authors:** Fei Han, Weiwei Wang, Meng Shi, Hao Zhou, Yiwen Yao, Caiyun Li, Anquan Shang

**Affiliations:** ^1^ Department of Laboratory Medicine, The Fourth Affiliated Hospital of Nanjing Medical University, Nanjing, China; ^2^ Department of Laboratory Medicine, The Second People’s Hospital of Lianyungang & The Oncology Hospitals of Lianyungang, Lianyungang, China; ^3^ Department of Cardiothoracic Surgery, Huashan Hospital, Fudan University, Shanghai, China; ^4^ Department of Internal Medicine V-Pulmonology, Allergology, Respiratory Intensive Care Medicine, Saarland University Hospital, Homburg, Germany; ^5^ Department of Laboratory Medicine, Pukou Branch of Jiangsu People’s Hospital & Nanjing Pukou District Central Hospital, Nanjing, China

**Keywords:** bacteria, extracellular vesicles, outer membrane vesicles, mechanisms of disease, respiratory tract diseases

## Abstract

Infectious diseases are the leading cause of death in both adults and children, with respiratory infections being the leading cause of death. A growing body of evidence suggests that bacterially released extracellular membrane vesicles play an important role in bacterial pathogenicity by targeting and (de)regulating host cells through the delivery of nucleic acids, proteins, lipids, and carbohydrates. Among the many factors contributing to bacterial pathogenicity are the outer membrane vesicles produced by the bacteria themselves. Bacterial membrane vesicles are being studied in more detail because of their potential role as deleterious mediators in bacterial infections. This review provides an overview of the most current information on the emerging role of bacterial membrane vesicles in the pathophysiology of pneumonia and its complications and their adoption as promising targets for future preventive and therapeutic approaches.

## Introduction

**Figure 1:**
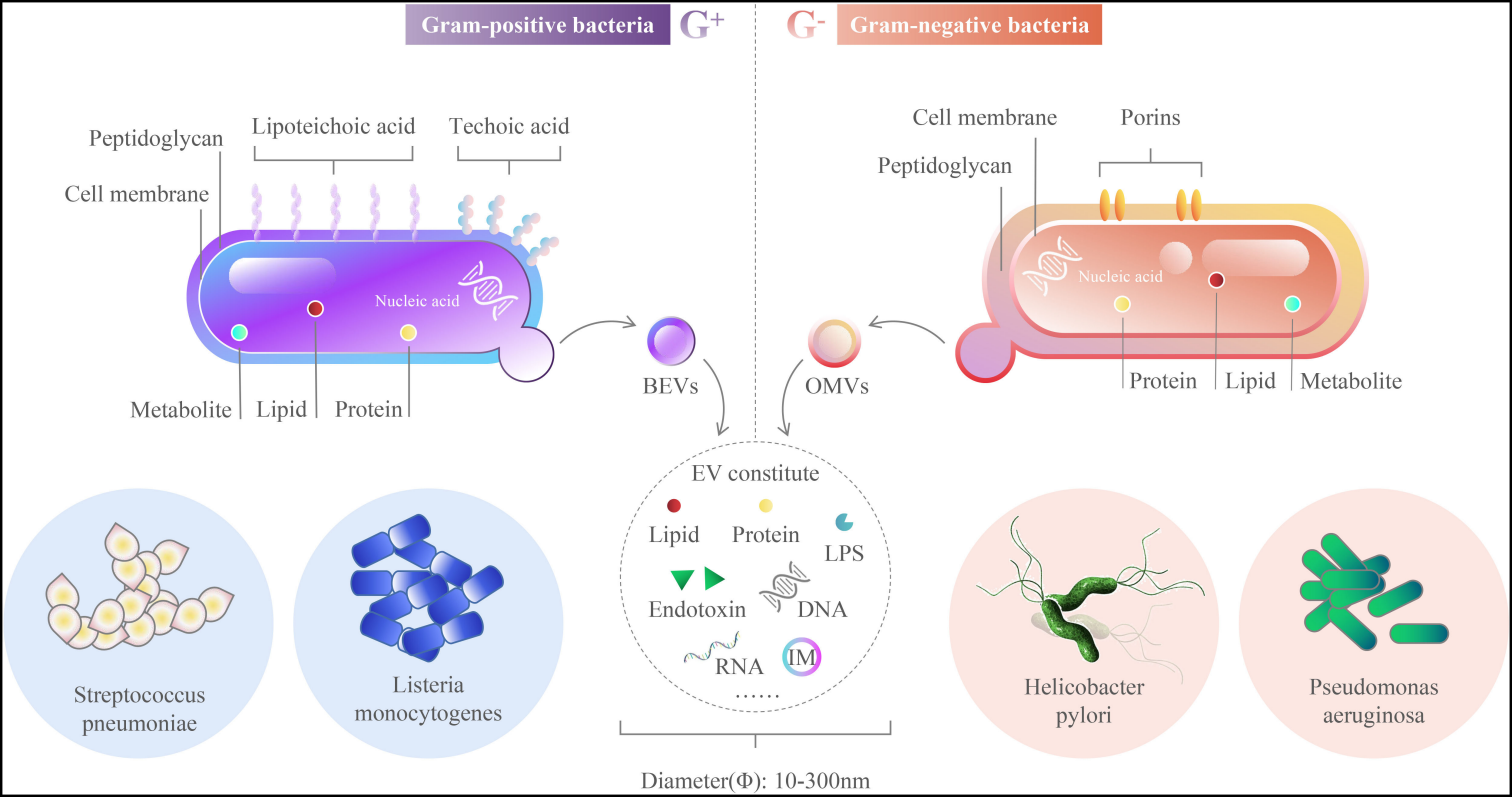
Outer Membrane Vesicles main components.

Both Gram-positive and Gram-negative bacteria produce extracellular vesicles (EVs), which are referred to as membrane vesicles (MVs) and outer membrane vesicles (OMVs), respectively, based on their proposed mechanisms of release. For example, OMVs are thought to bleb from the outer membrane of Gram-negative bacteria and as a result, encapsulate periplasmatic content, whereas MVs are thought to bud from the cytoplasm (Ñahui Palomino et al., 2021). OMVs resemble mammalian-derived EVs in size and are expected to promote bacterial-host communication by carrying a range of bioactive substances such as proteins, nucleic acids, lipids, and metabolites. OMVs are associated with bacterial-bacterial and bacterial-host interactions, promote health, or cause a variety of diseases. Vesicles are about 10 to 300 nm in diameter, originate from the outer membrane (OM), and are comprised of lipids, proteins, lipopolysaccharides (LPS), phospholipids, DNA, RNA, and the inner membrane (IM) (**Figure 1**) **(** Furuyama and Sircili, 2021). The passage components of OMVs into have cells are assorted and have not been totally explained due to the species, glue particles and their items. As a general rule, the OMVs are shipped into the cells through endocytosis. In endocytosis, film spaces invaginate, trailed by being squeezed off from the inward side of the cell layer and moved inside the cell. Likewise, some OMVs are recommended to meld with lipid pontoons in the film and the items are delivered into the cytoplasm.

OMVs have the potential to initiate inflammatory reactions in response to certain infections, such as *Streptococcus pneumoniae*, *Pseudomonas aeruginosa*, and *L. pneumophila (* Furuyama and Sircili, 2021). These bacteria are thought to be responsible for lung inflammation by triggering immunological responses in the respiratory epithelium. This has led some scientists to believe that OMVs can actually boost the immune system. When pathogenic bacteria break their outer membrane, they produce OMVs, which play a crucial role in the interaction between the host and the pathogen. These include establishing colonizing ecological niches, delivering virulence factors to host cells, forming bacterial communities (biofilms), and functioning as decoys for bacterial evasion of immunity and evasion of the host immune system (Cui et al., 2021).

## Streptococcus pneumoniae

The bacterium known as *Streptococcus pneumoniae* is responsible for a considerable quantity of illness and death all across the globe (Codemo et al., 2018). Every year, pneumococcal infections claim the lives of around one million young children under the age of five all over the globe (Henriques-Normark and Tuomanen, 2013). Pneumococci are a key contributor to the development of community-acquired pneumonia, sepsis, and meningitis; nevertheless, they are also a substantial contributor to the development of less severe respiratory infections such as otitis media and sinusitis (Codemo et al., 2018; Xiong et al., 2019).

### OMVs reduce pneumococcal-modulated phagocytosis by strongly binding components of the complement system

*Streptococcus pneumoniae* strain TIGR4 OMVs are high in lipoproteins and short-chain fatty acids, and they carry a wide range of surface proteins and toxins (hemolysins) (Yerneni et al., 2021). OMVs generated a rise in the production of pro-inflammatory cytokines in A549 lung epithelial cells as well as in human monocyte-derived dendritic cells, including interleukin 6 (IL-6), IL-8, IL-10, and tumor necrosis factor (TNF). OMVs induced high expression of the four cytokines, but they were unable to trigger the release of IL-1 or IL-12 into the supernatant (Codemo et al., 2018; Vitse and Devreese, 2020). The study also examined the ability of MV to regulate the release of interleukins from DCs and found increased TNF-α secretion by DC2.4 cells after exposure to streptococci MV, a slight increase in IL -10 production by pneumococcal OMVs treated DC cells, and no change in IL -12 secretion by OMVs treated DC2.4 cells, an observation consistent with previously reported results (Mehanny et al., 2020). On the other hand, pneumococcal OMVs readily binds factor H (FH) from human serum in a PspC-dependent manner; this factor is a negative regulator of the bypass route (Yuste et al., 2010; Codemo et al., 2018). First, FH increases the factor I-dependent cleavage of C3b that is attached to the surface of the bacterial cell, which results in the production of iC3b, which speeds up the degradation of C3b. Second, FH separates B factor from the bypass C3 convertase C3bBb, which decreases the amount of C3b that is deposited on bacteria (Yuste et al., 2010; Hyams et al., 2013). Third, FH inhibits the production of C3 convertase C3bBb on the surface of bacteria by preferentially binding C3b and preventing C3b from attaching to factor B (FB) (Quin et al., 2006; Quin et al., 2007; Li et al., 2007).

**Figure 2:**
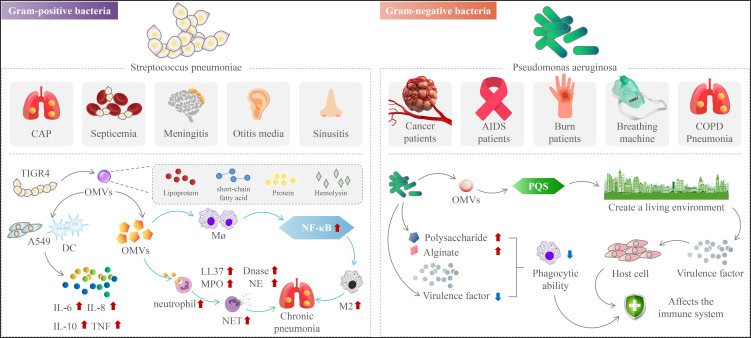
The mechanism of action of outer membrane vesicles in chronic respiratory diseases.

In conclusion, OMVs reduce pneumococcal conditioning phagocytosis by strongly binding components of the complement system. The capacity of OMVs to activate the alternative route (M2) shows that OMVs enhance pneumococcal chronicity. The OMVs stimulate significant NF-kB signaling in macrophages in a dose-dependent manner, and they differentiate human macrophages into the (M2) phenotype (Yerneni et al., 2021). OMVs are classified as pneumococcal immune regulators. They influence immune cell recruitment and cytokine production to influence the adaptive immune response (Yerneni et al., 2021). Our findings indicate that OMVs are incorporated into host cells after pneumococcal infection. OMVs help the host defend itself by inducing immune cell recruitment and cytokine responses. On the other hand, by creating an anti-inflammatory milieu for bacterial survival, these OMVs contribute to pneumococcal chronicity infected (**Figure 2**) **(** Yerneni et al., 2021). As a consequence, we hypothesize that OMVs are critical effectors of the nuanced interactions between bacteria and host that decide whether an infection is cleared, carried, or transmitted.

### NET released by degraded human neutrophils

Pneumococcal virulence factors that facilitate host cell adhesion and/or invasion and/or antagonize the immune system have been found in studies. Several pneumococcal proteins, for example, have been linked to complement-mediated immunity evasion (Jhelum et al., 2018). The alveolar macrophage is the first kind of immune cell that engages in the fight against *Streptococcus pneumoniae* during the early stages of an infection (Jambo et al., 2010). As the lung infection progresses, pneumococci attract neutrophils, which are essential for bacterial clearance. Human bacterial pathogens such as Streptococcus pneumoniae are phagocytosed by neutrophils and killed by various oxidative and/or nonoxidative mechanisms. Neutrophils employ various strategies, such as the release of antimicrobial peptides and the production of reactive oxygen species, to combat these pathogens (**Figure 2**) **(** Winterbourn et al., 2016). NETs consist of chromatin adorned with antimicrobial peptides such as LL37, neutrophil elastase (NE), myeloperoxidase (MPO), defensins, etc (Cortjens et al., 2017). It has been reported that the formation of NET is mainly stimulated by various pathogens (von Köckritz-Blickwede et al., 2016). Examples include *Neisseria meningitidis (* Lappann et al., 2013), *Pseudomonas aeruginosa (* Rada et al., 2013), *Staphylococcus aureus (* Malachowa et al., 2013), *Mycobacterium tuberculosis (* Dang et al., 2018), *Streptococcus pyogenes (* Buchanan et al., 2006), and *Burkholderia pseudomallei (* de Jong et al., 2014), *Dengue virus (* Sung and Hsieh, 2021), and in mice that had pneumococcal infection, signs of *Aspergillus* were found in the lungs (Rafiq et al., 2022).

Secretion of OMVs-associated DNase by pneumococci was shown to degrade neutrophil- released NET and its absence reduced virulence of Streptococcus pneumoniae in a mouse model of sepsis, suggesting that OMVs-associated DNase is involved in bypassing the host innate immune system (Beiter et al., 2006). At least two DNases were found to be present in Streptococcus suis. Wild-type Vibrio cholerae rapidly degrades DNA components of NET through the combined activity of two DNases (Dns and Xds) (Jhelum et al., 2018). Secreted DNase contribute to the degradation of NET DNA and protects *P. aeruginosa* from NET-mediated killing (Seper et al., 2013).

## Pseudomonas aeruginosa *(P. aeruginosa)*

*P. aeruginosa* is an opportunistic Gram-negative bacterium that attacks immunocompromised hosts. These immunocompromised hosts include cancer and AIDS patients, burn victims, and people on ventilators. *P. aeruginosa* releases OMVs, a method of interacting with hosts and microbes in their natural environment (Wilton et al., 2018). In addition to providing virulence factors and toxins to host cells, OMVs also triggers Pseudomonas quinolone signaling (PQS) (**Figure 2**). This process allows bacteria to develop a colonial ecological niche by selectively killing or promoting the growth of different types of bacteria (Kadurugamuwa and Beveridge, 1995; Kuehn and Kesty, 2005; Nakamura et al., 2008; Bomberger et al., 2009; Tashiro et al., 2012; Cooke et al., 2019; Cooke et al., 2020).

In recent years, it has been shown that the majority of pro-inflammatory host immune responses induced in the lung by pathogen-associated molecular patterns (PAMP) are mediated by OMVs. These nanoparticles, which are discharged in huge amounts into the bronchial lumen, include vital virulence components, among other things. PAMPs such as lipopolysaccharide (LPS), peptidoglycan (PG), flagellin (Flag), pore proteins (Por), and lipoproteins (Lip) bind to Toll-like receptors (TLR) in host cells, which signal *via* mitogen-activated protein kinase (MAPK), resulting in the release of pro-inflammatory cytokines and IL -8 in human airway epithelial cells (Saatian et al., 2006; Zhong and Kyriakis, 2007).

Cytokine release draws neutrophils and macrophages to infection sites, clearing bacteria. *P. aeruginosa* uses multiple ways to evade the human immune system to develop persistent infection. These techniques include upregulating polysaccharide and alginate synthesis, downregulating virulence factor expression, reducing phagocytic absorption of *P. aeruginosa* by immune cells, and eliminating flagellar mobility.

### Regulation of virulence factor

Bacterial toxicity is the main obstacle to the use of bacteria or their derivatives. Mechanisms mediated by OMVs may also suppress the host immunological response to *P. aeruginosa*. For example, Cif (PA2934), a virulence factor in OMVs, reduces USP10-mediated deubiquitination of CFTR by inactivating host cell deubiquitinating enzyme (USP10) and increases CFTR degradation in lysosomes, which inhibits chloride secretion and thus reduces the ability to clear respiratory pathogens through mucus cilia, an important component of the pulmonary innate immune response (**Figure 2**) **(** Bomberger et al., 2011). Cif diminishes the adaptive immune response to viral infection, and thus reduces viral and bacterial clearance in the lung, primarily by decreasing TAP1, leading to a significant decrease in peptide transport to the ER, and inhibiting MHC class I-mediated viral and bacterial antigen presentation (Bomberger et al., 2014). Therefore, we hypothesize that the combined actions of the *P. aeruginosa* Cif virulence factors promote various microbial infections in the lungs of individuals who suffer from cystic fibrosis, chronic obstructive pulmonary disease, and bronchiectasis. To address this escape mechanism, researchers found that filipin III is a compound that disrupts cholesterol-rich lipid rafts in the parietal membrane of airway epithelial cells, reduces OMVs fusion with CFBE cells, and prevents OMV-associated Cif delivery to human bronchial epithelial cells (Stanton, 2017). HPβCD and MβCD reduce OMVs inhibition by disrupting cholesterol-rich lipid rafts Phe508del CFTR Cl-secretion because cyclodextrins have many non-specific effects, including removing cholesterol from raft and non-raft domains of the plasma membrane and altering cholesterol distribution between the plasma membrane and intracellular membranes (Barnaby et al., 2019). They also observed that HPβCD and MβCD reduced the planktonic growth and biofilm formation of *P. aeruginosa*. These observations are consistent with previous studies indicating that cyclodextrins inhibit population induction of Gram-negative bacteria, which is a key factor in biofilm formation and bacterial resistance to antibiotics in CF lungs (Morohoshi et al., 2013).

*P. aeruginosa* was treated with an inhibitory concentration of tobramycin (1 μg/ml) or an untreated control PAO1 strain and examined the effect of tobramycin on OMVs protein content using a liquid chromatography-tandem mass spectrometry (LC-MS/MS) method. They discovered that tobramycin decreased the abundance of several virulence-associated proteins (including AprA) in OMV and attenuated the inhibitory effect of OMV on the secretion of Phe508del CFTR Cl - by VX-809-stimulated CF bronchial epithelial cells. In addition, they found that tobramycin inhibited the growth of CF bronchial epithelial cells. The host cells were killed by the alkaline protease AprA, which also suppressed the host’s cellular and humoral immunological responses (Lee et al., 2018). They came to the conclusion that the elimination of AprA, AlpA, AlpD, and AlpE as well as other virulence determinants in tobramycin-induced OMV might enhance lung function and minimize lung damage, delivering a favorable therapeutic effect with only small reductions in bacterial burden (Koeppen et al., 2019).

### Regulating vitronectin (VN)

In addition to utilizing modulators of the alternative and classical/agglutinin pathways, many microorganisms acquire human end metabolic pathway inhibitors, VN, to escape the immune system (Zipfel et al., 2013). Hic interacts with VN and FH, which may lead to *Streptococcus pneumoniae* serotype 3 colonization and invasive illness (Kohler et al., 2015). Recent studies have shown that endotoxin in *P. aeruginosa* OMVs induces the release of VN into the bronchoalveolar space, thereby protecting against complement-mediated killing. VN is a 75 kDa glycoprotein found in plasma and extracellular matrix and belongs to this class of complement regulators. The glycoprotein binds to C5b-7 at sub-stable membrane-bound sites, thereby inhibiting membrane insertion of the complex, but can also directly inhibit C9 polymerization (Podack et al., 1984). VN consists of several structural domains, including TGF-β structural domain, the integrin receptor binding motif Arg-Gly-Asp (RGD), the heme-binding protein-like structural domain, and three heparin-binding structural domains (HBD). VN is released into the blood by hepatocytes but may also be generated by respiratory epithelial cell**s** (Salazar-Peláez et al., 2015). It prevents self-injury by blocking membrane attack complex formation (Sheehan et al., 1995). VN is also related with inflammatory processes, as shown by elevated glycoprotein levels in chronic lung disease patients (Carpagnano et al., 2003; Hallström et al., 2016). By binding Perlins to surface proteins, microorganisms suppress membrane attack complexes and achieve complement resistance. Surface-bound VN may promote bacterial adherence to the epithelium by boosting bacterial-host cell-cell interactions. *P. aeruginosa* from the bronchoalveolar area demonstrated improved boronin binding capability, indicating boronin-dependent pathogenicity in the lung (Paulsson et al., 2015). The researchers found that NTHi and *P. aeruginosa* cells were 3.9-fold and 2.6-fold more viable when compared to BALF preincubation with and without bollenin (Paulsson et al., 2018). These studies indicate that bacterial cells acquire BALF VN to avoid complement-mediated lysis and remain in mammalian hosts. This work uncovers a complicated host-pathogen interaction in which the innate immune system detects bacteria and their OMVs, reacts to them, and gives the bacteria tools to avoid the complement system’s antimicrobial effects.

### Secretion of small RNA (sRNA)

The fact that many bacterial regulatory small RNAs (sRNAs) have multiple mRNA targets places them at the center of regulatory networks that assist bacteria in adjusting to changes in their surrounding environment (Bossi and Figueroa-Bossi, 2016). OMVs and BEVs mediate the transfer of sRNA and tRNA fragments between bacterial and mammalian cells without direct contact. sRNA52320 was transferred from OMVs to host cells, resulting in a decrease in OMV-stimulated IL -8/KC production of human airway epithelial cells and mouse lungs and a decrease in neutrophil infiltration in an animal model exposed to OMVs. sRNA52320 mostly targets mRNAs in the TLR2/4-induced innate immune response pathway, whereas other receptors and pathways remain unaffected (Koeppen et al., 2016). Similarly, differently packaged sncRNAs were found in *H. pylori* OMVs, and results indicated that sncRNAs (sR-2509025 and sR-989262) were enriched for OMVs and inhibited LPS or OMV-induced IL-8 production from cultured AGS cells (Zhang et al., 2020). MicroRNA-sized RNA fragments identified in periodontopathogenic OMVs reduced IL-5, IL-13 and IL-15 secretion in lymphocyte Jurkat T cells (Choi et al., 2017). In addition, transfer of periodontal pathogen OMVs exRNA to the brain may contribute to neuroinflammatory diseases such as Alzheimer’s disease (Han et al., 2019). The sRNA in OMVs secreted by *E. coli* is transferred to bladder epithelial cells and inhibits LPS-induced IL-1a secretion (Dauros-Singorenko et al., 2020). OMVs secreted by Listeria monocytogenes with sRNA rli32 promotes intracellular growth of the pathogen by relying on RIG-I (retinoic acid-inducible gene I) and stimulating the production of IFN-β by bone marrow-derived macrophages (Frantz et al., 2019). During Listeria monocytogenes infection, high levels of type I IFN antagonize IFN-γ signaling by downregulating interferon γ receptors (IFNGR) on antigen-presenting cells (APCs) (Rayamajhi et al., 2010), increase lymphocyte apoptosis (O'Connell et al., 2004), enhance macrophage cell death (Stockinger et al., 2002), reduce protective interleukin 12 (IL-12) and tumor necrosis factor α (TNF-α) production (Auerbuch et al., 2004), and inhibit neutrophil migration (Brzoza-Lewis et al., 2012), thereby contributing to bacterial growth. The microenvironment for bacterial growth was created by increasing lymphocyte apoptosis, enhancing macrophage death, reducing protective interleukin 12 (IL-12) and tumor necrosis factor α (TNF-α) production, and inhibiting neutrophil migration (Osborne et al., 2017).

### Inhibition of major histocompatibility complexes (MHC)

It has been postulated that OMVs directly or indirectly influence gene expression in target cells through host immune receptor signaling. The presentation of antigen is critical for the immune response (Armstrong et al., 2020). MHC molecules communicate peptides to other immune cells in order to activate adaptive immunological responses. Communication between antigen-presenting cells (APCs) and T lymphocytes is required for the host response to bacterial infection. This message is provided to helper T cells by MHC class II antigen presentation, which is followed by an adaptive T cell or B cell response. The interaction between T cells and MHC II, as well as their surroundings, is crucial to the phenotypic and effectiveness of the inflammatory response to infection.

Macrophages are important APCs in the lung because of their immunological flexibility and capacity to perceive and adapt to the local microenvironment (Xing et al., 2020). Pulmonary macrophages are important in the identification and clearance of germs, as well as in the polarization of innate and adaptive immunity. OMVs from the common Cystic Fibrosis (CF) bacteria *P. aeruginosa* were found for the first time to decrease the production of MHC-related markers in human lung macrophages (Armstrong et al., 2020). A study discovered that OMVs lowered the expression of nine distinct MHC II-associated genes, including HLA-DRA, -DRBs, -DMB, -DPs, CD74, CD9, and CTSS. HLA-DRA and -DRB are MHC II complex heterodimeric components that bind antigen fragments processed inside phagocytosed lysosomes. HLA-DMB is the enzyme responsible for removing CLIP from the HLA-DR cleft, allowing antigen fragments to bind. HLA-DP is a paralog of the HLA class II beta chain. CD74 is largely recognized as an invariant chain of MHC class II and plays a role in the molecular processing of MHC class II by the Golgi, as well as being a crucial component in the functional presentation of MHC class II restricted antigens (Roche and Furuta, 2015). CD9 controls MHC class II trafficking throughout the cell. CTSS is histone S, a cysteine protease found in the lysosome that degrades CD74 and pathogenic peptides. All of these components work together to process and deliver MHC class II antigens to CD4^+^ T cells, acting as a link between innate and adaptive immunity during pathogen infection. This shows that *P. aeruginosa* OMVs, independent of chromosomal location, contain a variety of components that selectively target MHC molecular inhibition as an immune evasion strategy.

### Altered methylation

*P. aeruginosa* OMVs causes dysregulation of the appropriate immune response to infection in macrophages by altering DNA methylation in human lung macrophages (Kyung Lee et al., 2021). Epigenetic modifications alter the pattern of gene expression and have been reported to be important during the innate immune response, regulating B-cell fate and function and controlling T-cell differentiation and memory responses (Lau et al., 2018; Zhang and Cao, 2019; Zhang and Good-Jacobson, 2019).

Gastric cancer is a disease that has been identified to be caused by epigenetic modifications following bacterial infection (Maekita et al., 2006). Alterations in the epigenome, including histone modifications and DNA methylation, are believed to be crucial activating or inhibitory factors, which raises the potential that fast alterations in DNA methylation play a role in the innate immune response (Marr et al., 2014; Morales-Nebreda et al., 2019; Qin et al., 2021). It is important to note that viruses have the potential to change the methylation of DNA and/or impact the expression and activity of DNA methylation modifiers like TET and DNMT. This may lead to changes in the expression of important host genes that are involved in immune response. These two groups of proteins are directly engaged in the mechanism of DNA methylation: DNMTs induce and maintain DNA methylation, whereas TETs catalyze demethylation *via* a series of stages. Infection of dendritic cells by Mycobacterium tuberculosis results in rapid loss of DNA methylation of distal enhancers of major immune transcription factors, including NF-kB and members of the interferon regulatory factor family, within 24 h of activation. This suggests that DNA methylation controls the innate immune response. DNA methylation actively blocks the binding of certain transcription factors (TFs) to the promoter, thereby impairing transcription. During development, activation, and tumor transformation, all three TETs contribute to dynamic demethylation, which is linked with significant transcriptional reprogramming in cells (Qin et al., 2021). During bacterial and viral infections, respectively, TET2 and TET3 have been demonstrated to decrease the expression of proinflammatory cytokines by bringing HDAC1/2 to the promoters of cytokine-encoding genes (Zhang et al., 2015; Xue et al., 2016; Fuster et al., 2017). TET2 also promotes the production of anti-inflammatory cytokines. TET2 also promotes the recruitment of the multicomb repressor complex 2 to the promoters of CpG dinucleotide-rich genes, which results in transcriptional repression (Wu et al., 2011). TET1 may bind to the SIN3A co-repression complex, resulting in a 5hmC-independent transcriptional effect (Williams et al., 2011), which might be a mechanism for TET1-mediated IL-1B transcriptional repression (Neves-Costa and Moita, 2013).

Aberrant DNA methylation in sepsis-related monocytes is linked to inflammatory cytokines and organ failure (Lorente-Sorolla et al., 2019). Within twenty-four hours of the activation of distal enhancers of important immune transcription factors, such as NF-kB and members of the interferon regulatory factor family, Mycobacterium tuberculosis infection of dendritic cells causes a rapid loss of DNA methylation. This loss occurs as a direct result of the infection. According to this, DNA methylation is likely responsible for regulating the innate immune response (Pacis et al., 2015). DNA methylation may also affect the release of gingival cytokines, which in turn affects how the immune system reacts to *Porphyromonas gingivalis (* Drury and Chung, 2015; Jurdziński et al., 2020).

DNA methylation suppresses transcriptional processes in mature CD4^+^ T cells in neonates with pneumonia, and these results imply that DNA methylation might serve as a therapeutic target for pediatric lung (McGrath-Morrow et al., 2018). For example, altered DNA methylation status of the Igf2 gene in mouse placental tissue has been associated with maternal infection with the peripheral pathogen Campylobacter. These results are consistent with immune evasion strategies employed by other microorganisms in host-pathogen interactions that may lead to altered innate immune responses.

### Remodeling biofilm

Biofilm growth patterns are dominant in natural and disease environments, with over 65% of infections estimated to be biofilm related (Potera, 1999). Planktonic bacteria first attach to a surface, thus transforming into a biofilm lifestyle (Sauer et al., 2002). Afterwards, they produce an extracellular polymer (EPS) that envelops the bacteria and protects them from the environment. the EPS consists of polysaccharides, extracellular DNA, proteins and lipids, and OMVs (Hu et al., 2020). Recently, Schooling and Beveridge reported that bacteria with OMVs present in the biofilm matrix and biofilm of *P. aeruginosa* have higher OMVs synthesis rates than when cultured in planktonic environments (Schooling and Beveridge, 2006). The production of PaAP is controlled by a process known as population sensing (QS), and the protein is released through the Pseudomonas aeruginosa type II Xcp secretion pathway (Michel et al., 2007). Production of PaAP in *P. aeruginosa* leads to an increase in the activity of an endogenous protease that targets secreted OMVs. This in turn leads to OMV-induced cell separation and contributes to the remodeling of the overall biofilm structure. OMV-induced changes eventually lead to an increase in the Psl/biomass ratio in the early biofilm matrix, which helps to protect growing colonies from the deleterious effects of antimicrobial agents (Esoda and Kuehn, 2019). Based on these findings, there is a possibility that PaAP plays a role in fine-tuning pathogenesis, including biofilm production and infection. Proteomic investigation of *P. aeruginosa* biofilms confirmed that proteins associated with Membrane Vesicles make up more than 20% of the total matrix proteome. enzymes involved in the transport of small molecules, iron absorption, and antibiotic resistance were among the proteins shown to be related with OMVs (Couto et al., 2015). Additionally, vesicles isolated from late P. aeruginosa biofilms were richer in drug-binding proteins, which increased the antibiotic resistance of bacterial species inside these biofilms. It was further shown that MV secreted by *P. aeruginosa* is controlled by a population-sensing system and provides extracellular DNA (eDNA) and LPS to the forming biofilm (Nakamura et al., 2008). Furthermore, studies on *P. aeruginosa* biofilms have shown that OMVs secreted by one species is capable of lysing neighboring bacteria to release nutrients as a source of growth and to release eDNA for biofilm construction (Beveridge et al., 1997). However, subsequent studies have shown that OMVs itself is actually incorporated into the biofilm matrix (Schooling and Beveridge, 2006). In addition to this, 11 proteins linked with antimicrobial resistance were shown to be connected with OMVs. It was discovered that all of the proteins that are encoded by the vanA cluster of vancomycin-resistant strains are related with MV. This suggests that bacteria are able to employ Enterococcus faecalis OMVs to release proteins that increase virulence, pathogenicity, and antibiotic resistance (Schooling and Beveridge, 2006). OMVs are a factor in the creation and maintenance of biofilms. The discovery that enterococcal virulence factors AtlA, Esp, and SagA, all of which contribute to the production of enterococcal biofilm, are connected with OMVs may imply that E. coli may be able to produce OMVs. faecium OMVs have the potential to contribute to the production of biofilms (Kropec et al., 2011; Paganelli et al., 2015; Wang et al., 2015). The study investigated the production and functional activity of OMVs in surface-associated microbial communities or biofilms of the fungal pathogen Candida albicans. Biofilm vesicle cargoes include ESCRT subunits Hse1 and Vps27, and most ESCRT-deficient mutations result in reduced biofilm EVs production, reduced levels of matrix polysaccharides, and greatly increased susceptibility to the antifungal drug fluconazole. Exogenous administration of OMVs restores the biofilm resistance phenotype and matrix composition. The OMVs of the biofilm may deposit substances that directly contribute to the structure of the matrix, and they may also have catalytic activity involved in polysaccharide synthesis of the matrix (Zarnowski et al., 2018). Biofilm cells release EVs that promote extracellular matrix formation and resistance to antifungal drugs (Zarnowski et al., 2021).

It was also found that the release of OMVs, which contain two chromosomally encoded β-lactamases, increases when Stenotrophomonas maltophilia infection is treated with β-lactam antibiotics. They demonstrate the ability of these β-lactamase-packed OMVs to establish extracellular β-lactam degradation. The investigation also reveals that the cohabitating species *P. aeruginosa* and *Burkholderia cenocepacia* have significantly higher apparent MICs for imipenem and ticarcillin.

## Summary

Lung infection-associated bacteria pose a rising hazard to worldwide public health. OMVs are outer membrane vesicles that are secreted by Gram-negative bacteria. These OMVs have the ability to transfer infectious agent into the cytoplasm of the host cell, which induces a protective immune response in the body. This opens up a new avenue for the potential reduction of tissue damage caused by immune tolerance. Consequently, it is crucial to shed more insight on the techniques these bacteria use to improve their pathogenicity. The significance of OMVs in immune evasion is highlighted. By targeting OMV-related components implicated in the interaction of these vesicles with human lung cells or macrophages, new treatment approaches for these infections may become available. In addition, the immunomodulatory properties of OMVs might be used to develop vaccines that protect patients from bacterial infections.

## Author contributions

Writing—Original Draft: CL and AS; Figure design: HZ; Data collection: MS and FH; Searching: YY and AS; Review & Editing: All authors. All authors read and approved the final manuscript.
